# Encapsulation of Upconversion Nanoparticles in Periodic Mesoporous Organosilicas

**DOI:** 10.3390/molecules24224054

**Published:** 2019-11-09

**Authors:** Saher Rahmani, Chiara Mauriello Jimenez, Dina Aggad, Daniel González-Mancebo, Manuel Ocaña, Lamiaa M. A. Ali, Christophe Nguyen, Ana Isabel Becerro Nieto, Nadège Francolon, Erwan Oliveiro, Damien Boyer, Rachid Mahiou, Laurence Raehm, Magali Gary-Bobo, Jean-Olivier Durand, Clarence Charnay

**Affiliations:** 1 Institut Charles Gerhardt Montpellier, case 1701, UMR5253, CNRS-UM-ENSCM, Place Eugène Bataillon, 34095 Montpellier, CEDEX 05, France; rahmeni.sahar@yahoo.fr (S.R.); chiaramauriellojimenez@gmail.com (C.M.J.); erwan.oliveiro@umontpellier.fr (E.O.); laurence.raehm@umontpellier.fr (L.R.); durand@univ-montp2.fr (J.-O.D.); 2 Institut des Biomolécules Max Mousseron UMR 5247 CNRS, UM-Faculté de Pharmacie, 15 Avenue Charles Flahault, 34093 Montpellier, CEDEX 05, France; dina.aggad@gmail.com (D.A.); lamiaa.ali@umontpellier.fr (L.M.A.A.); christophe.nguyen@umontpellier.fr (C.N.); magali.gary-bobo@inserm.fr (M.G.-B.); 3 Instituto de Ciencia de Materiales de Sevilla (CSIC-US), c/Américo Vespucio, 49, 41092 Seville, Spain; dagoman_89@hotmail.com (D.G.-M.); mjurado@icmse.csic.es (M.O.); anieto@icmse.csic.es (A.I.B.N.); 4 Université Clermont Auvergne, CNRS, SIGMA Clermont, ICCF, F-63000 Clermont–Ferrand, France; francolon.nadege@hotmail.fr (N.F.); damien.boyer@sigma-clermont.fr (D.B.); rachid.mahiou@uca.fr (R.M.)

**Keywords:** upconversion, periodic mesoporous organosilica nanoparticles, cancer

## Abstract

(1) Background: Nanomedicine has recently emerged as a promising field, particularly for cancer theranostics. In this context, nanoparticles designed for imaging and therapeutic applications are of interest. We, therefore, studied the encapsulation of upconverting nanoparticles in mesoporous organosilica nanoparticles. Indeed, mesoporous organosilica nanoparticles have been shown to be very efficient for drug delivery, and upconverting nanoparticles are interesting for near-infrared and X-ray computed tomography imaging, depending on the matrix used. (2) Methods: Two different upconverting-based nanoparticles were synthesized with Yb^3+^-Er^3+^ as the upconverting system and NaYF_4_ or BaLuF_5_ as the matrix. The encapsulation of these nanoparticles was studied through the sol-gel procedure with bis(triethoxysilyl)ethylene and bis(triethoxysilyl)ethane in the presence of CTAB. (3) Results: with bis(triethoxysilyl)ethylene, BaLuF_5_: Yb^3+^-Er^3+^, nanoparticles were not encapsulated, but anchored on the surface of the obtained mesoporous nanorods BaLuF_5_: Yb^3+^-Er^3+^@Ethylene. With bis(triethoxysilyl)ethane, BaLuF_5_: Yb^3+^-Er^3+^ and NaYF_4_: Yb^3+^-Er^3+^nanoparticles were encapsulated in the mesoporous cubic structure leading to BaLuF_5_: Yb^3+^-Er^3+^@Ethane and NaYF_4_: Yb^3+^-Er^3+^@Ethane, respectively. (4) Conclusions: upconversion nanoparticles were located on the surface of mesoporous nanorods obtained by hydrolysis polycondensation of bis(triethoxysilyl)ethylene, whereas encapsulation occurred with bis(triethoxysilyl)ethane. The later nanoparticles NaYF_4_: Yb^3+^-Er^3+^@Ethane or BaLuF_5_: Yb^3+^-Er^3+^@Ethane were promising for applications with cancer cell imaging or X-ray-computed tomography respectively.

## 1. Introduction

Periodic mesoporous organosilica nanoparticles (PMON) have gained increasing attention for nanomedicine applications [1,2,3]. These nanoparticles are prepared from trialkoxypolysilylated organic precursors without the presence of any silica sources. The properties of these nanoparticles are very different from those of Mesoporous Silica Nanoparticles (MSN), which are at the forefront of bio-applications [4,5,6,7]. Indeed, PMON present higher loading capacities of hydrophilic and hydrophobic molecules than MSN due to the interaction with the organic matrix, and are less hemolytic than MSN as well as being biocompatible [8,9]. Degradability of PMON can be triggered by glutathione by introducing disulfide bridges in the matrix [10]. Core-shell systems have also been described with iron oxide [11], gold [12,13], or nanodiamonds [14] as the core. In the course of our work on PMON, we were interested in systems for theranostic applications with upconversion nanoparticles as their core, capable of being imaged by infrared or X-ray excitations. Indeed, upconversion nanoparticles present attractive features [15] such as near-infrared excitation at low power with deep penetration in living tissues, and biocompatibility in vivo when stabilized with dendrimers [16], or a mesoporous silica shell [17,18]. The matrix is usually NaYF_4_, but using Lu^3+^ instead of Y^3+^ endows the nanoparticles with X-ray sensitivity [19,20]. In the present work, we studied the encapsulation of upconversion NaYF_4_: Yb^3+^-Er^3+^ or BaLuF_5_: Yb^3+^-Er^3+^ nanoparticles with bis(triethoxysilyl)ethylene or bis(triethoxysilyl)ethane as the PMO precursors. We showed that no encapsulation occurred with BaLuF_5_: Yb^3+^-Er^3+^and bis(triethoxysilyl)ethylene. BaLuF_5_: Yb^3+^-Er^3+^ nanoparticles were anchored on the surface of BaLuF_5_: Yb^3+^-Er^3+^@Ethylene mesoporous nanorods, whereas core-shell systems were observed with bis(triethoxysilyl)ethane. We obtained core-shell nanoparticles with NaYF_4_: Yb^3+^-Er^3+^ or BaLuF_5_: Yb^3+^-Er^3+^as core. NaYF_4_: Yb^3+^-Er^3+^@Ethane nanoparticles were incubated with MCF-7 breast cancer cells and excitation in the NIR at 980 nm with a two-photon microscope made it possible to observe endocytosis of the nanoparticles. Alternatively, BaLuF_5_: Yb^3+^-Er^3+^@Ethane nanoparticles showed X-ray absorption properties when irradiated. Ethane-based PMON are, therefore, promising for theranostics applications.

## 2. Results and Discussion

**Figure 1:**
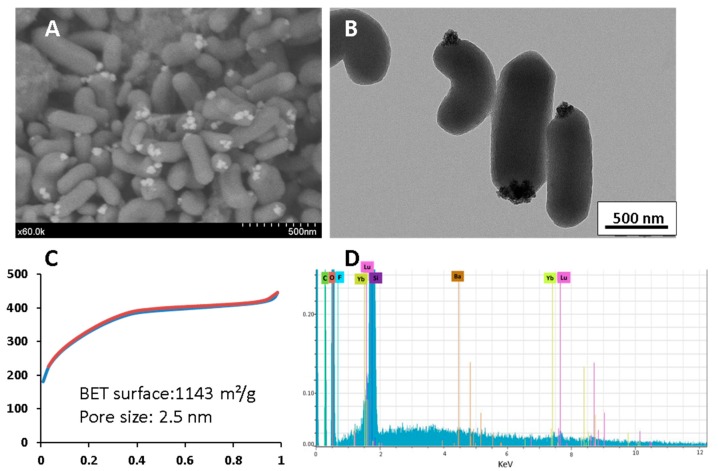
(**A**) SEM image of BaLuF_5_: Yb^3+^-Er^3+^@Ethylene (**B**) TEM of BaLuF_5_: Yb^3+^-Er^3+^@Ethylene, scale bar 500 nm. (**C**) N_2_ adsorption–desorption experiment of Yb^3+^-Er^3+^, BaLuF_5_ @Ethylene (**D**) EDX analysis of BaLuF_5_: Yb^3+^-Er^3+^@Ethylene. C, O, F, Yb, Lu, Si, Ba, Yb, Lu elements were detected.

For the synthesis of Yb^3+^-Er^3+^, BaLuF_5_@Ethylene, the sol-gel procedure was carried out in highly diluted conditions. First, CTAB was dissolved in water with NaOH and after the formation of micelles, BaLuF_5_: Yb^3+^-Er^3+^ nanoparticles were added in order to encapsulate the nanoparticles inside the micelles. Thirty minutes later, bis(triethoxysilyl)ethylene in EtOH was added and the mixture reacted for two hours. BaLuF_5_: Yb^3+^-Er^3+^@Ethylene nanoparticles were then centrifuged and the surfactant extracted with a solution of ammonium nitrate in 95% EtOH. The SEM and TEM of Yb^3+^-Er^3+^, BaLuF_5_@Ethylene nanoparticles are displayed in Figure 1A,B respectively. We observed 400 nm length and 150 nm width mesoporous nanorods as expected [21].

Surprisingly, the upconversion BaLuF_5_: Yb^3+^-Er^3+^nanoparticles were not embedded inside the structure, but were located on the surface, on the top of the nanorod, as clearly shown by SEM and TEM. The mesoporous structure was shown by N_2_ sorption experiments, with a BET surface of more than 1000 m^2^g^−1^ and small pores of 2.5 nm (Figure 1C). EDX confirmed the presence of characteristic elements of the inorganic matrix (Yb, Lu, Ba, and F).

**Figure 2:**
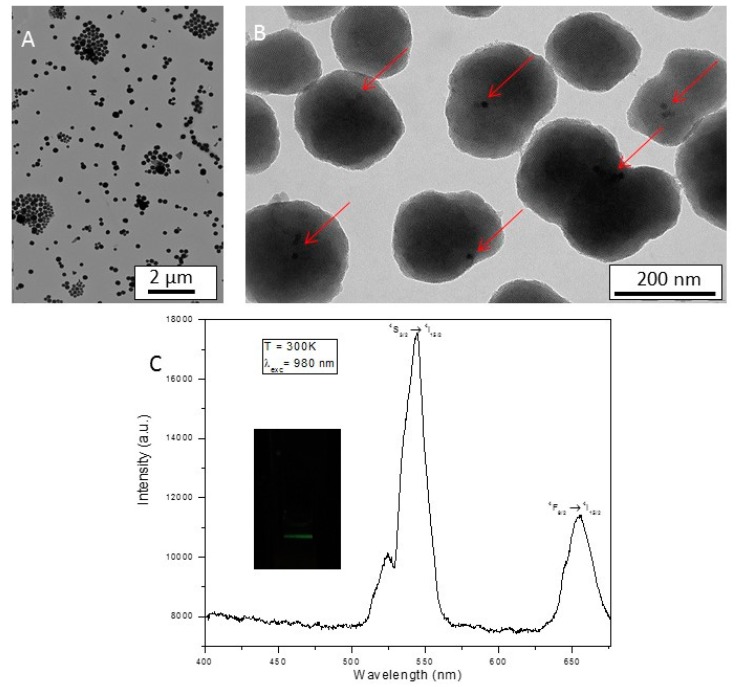

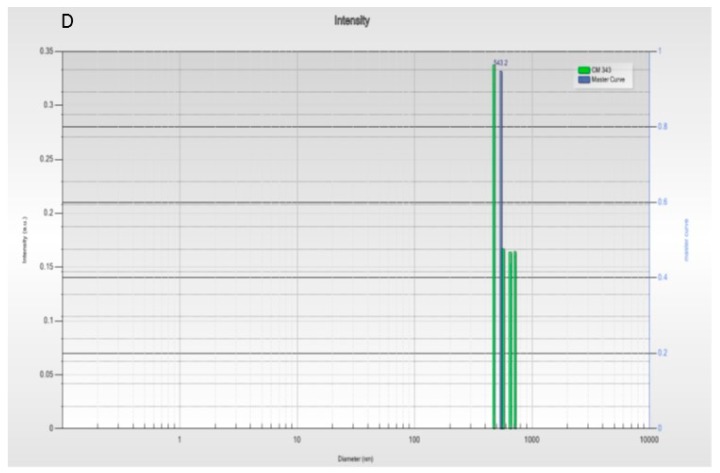
(**A**) TEM image of NaYF_4_: Yb^3+^-Er^3+^@Ethane scale bar 2 µm. (**B**) TEM of NaYF_4_: Yb^3+^-Er^3+^@Ethane, scale bar 200 nm. Red arrows show the embedded upconversion nanoparticles. (**C**) Luminescence spectrum of NaYF_4_: Yb^3+^-Er^3+^@Ethane upon excitation at 973 nm (**D**) DLS of NaYF_4_: Yb^3+^-Er^3+^@Ethane, in EtOH.

We then examined the hydrolysis-polycondensation of bis(triethoxysilyl)ethane in the presence of NaYF_4_: Yb^3+^-Er^3+^or BaLuF_5_: Yb^3+^-Er^3+^ nanoparticles, using essentially the same procedure as above. Note that bis(triethoxysilyl)ethane was not diluted in EtOH before its addition to the reaction. With NaYF_4_: Yb^3+^-Er^3+^, we obtained monodisperse 543 nm-diameter nanoparticles after reaction and extraction of the surfactant, as shown by TEM (Figure 2A) and dynamic light scattering (DLS) in EtOH, which displayed diameters centered at 543 nm showing the monodispersity of the system (Figure 2D). Magnification displayed core-shell systems with NaYF_4_: Yb^3+^-Er^3+^as the core and a mesoporous organosilica shell (Figure 2B). The core was still luminescent after encapsulation as shown in Figure 2C. After excitation at 980 nm, green (550 nm) and red (670 nm) luminescence was detected.

**Figure 3:**
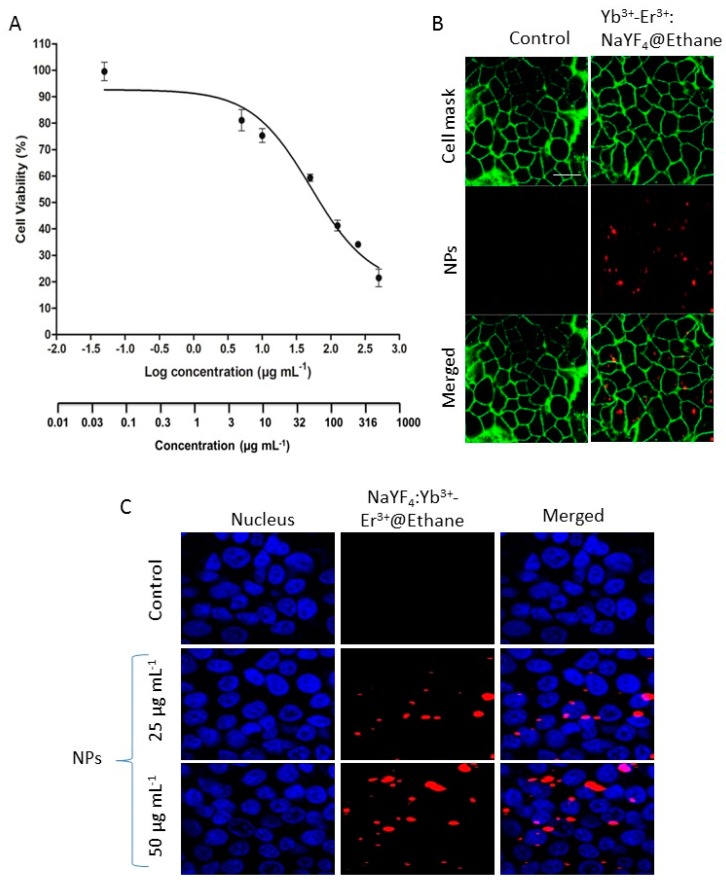
(**A**) Dose-response curve of MCF-7 cells treated with different concentrations of NaYF_4_: Yb^3+^-Er^3+^@Ethane for 3 days. Data are presented as mean ± SEM (n = 3). (**B**) Near-Infrared Imaging at 980 nm of MCF-7 cells incubated with cell mask (green) and NaYF_4_: Yb^3+^-Er^3+^@Ethane (red dots) at 50 µg mL^−1^. (**C**) Near-Infrared Imaging at 980 nm of MCF-7 cells incubated with Hoechst 33,342 (blue) and NaYF_4_: Yb^3+^-Er^3+^@Ethane (red dots) at 25 µg mL^−1^ or 50 µg mL^−1^.

We then decided to investigate the imaging potential of NaYF_4_: Yb^3+^-Er^3+^@Ethane and to study their cytotoxicity on cancer cells in culture. Cytotoxicity data in MCF-7 cells after 3 days of incubation with different concentrations of NaYF_4_: Yb^3+^-Er^3+^@Ethane showed the classical sigmoidal dose-response curves when plotted as a logarithmic function of nanoparticles concentration (µg mL^−1^) as shown in Figure 3A. The mean lethal concentration (LC_50_) value was 51.56 µg mL^−1^. NaYF_4_: Yb^3+^-Er^3+^@Ethane nanoparticles (50 µg mL^−1^) were then incubated with MCF-7 cancer cells, for 24 h. Fifteen minutes before the imaging experiment, the membranes of the cells were stained with a cell mask, and the cells were then imaged with a two-photon microscope at 980 nm. Figure 3B clearly demonstrated that NaYF_4_: Yb^3+^-Er^3+^@Ethane nanoparticles were endocytosed by MCF-7 cancer cells, as the luminescence of the system was detected inside the cells. In addition, the percentage of labeled cells (Figure 3C) with nanoparticles was determined using the ImageJ program on 9 pictures for each concentration. For a 25 µg mL^−1^ concentration, 46 ± 12% of cells were labeled with nanoparticles. Meanwhile, at a 50 µg mL^−1^ concentration, 58 ± 8% of cells were labeled with nanoparticles.

**Figure 4:**
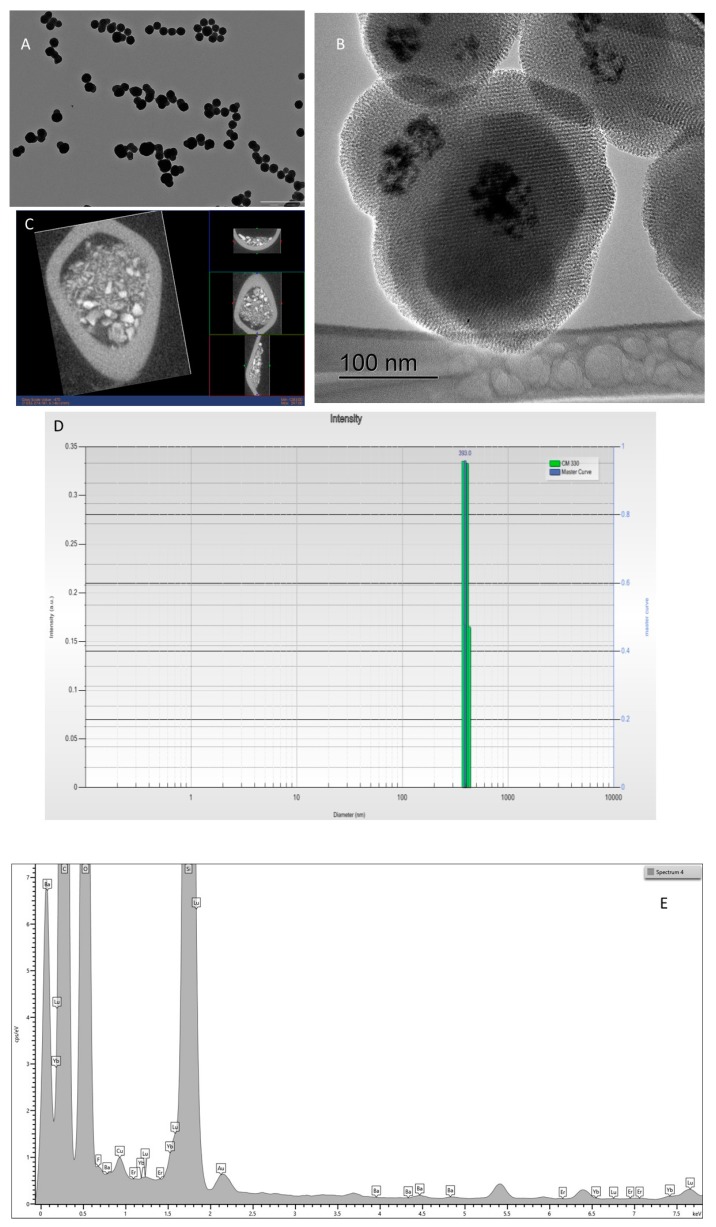
(**A**) TEM image of BaLuF_5_: Yb^3+^-Er^3+^@Ethane scale bar 1 µm (**B**) HRTEM of BaLuF_5_: Yb^3+^-Er^3+^@Ethane. (**C**) X-ray computed tomography of BaLuF_5_: Yb^3+^-Er^3+^@Ethane. (**D**) DLS of BaLuF_5_: Yb^3+^-Er^3+^@Ethane in EtOH. (**E**) EDX analysis of BaLuF_5_: Yb^3+^-Er^3+^@Ethane. Si, C, O, F, Ba, and Lu elements were detected.

With BaLuF_5_: Yb^3+^-Er^3+^nanoparticles, monodisperse core-shell systems were obtained with a size of 393 nm for BaLuF_5_: Yb^3+^-Er^3+^@Ethane as shown by TEM (Figure 4A). Moreover, Figure 4D shows monodisperse nanoparticles with diameters centered at 393 nm as displayed by DLS in EtOH. HRTEM revealed a mesoporous structure with a classical cubic arrangement of mesopores (Figure 4B) [22]. The BaLuF_5_: Yb^3+^-Er^3+^cores were wrapped with the periodic mesoporous organosilica shell. The presence of silicon, barium, lutetium, and fluoride elements are confirmed as observed on the displayed spectra (Figure 4E). Moreover, these nanoparticles were able to absorb X-ray, as shown by X-ray computed tomography (Figure 4C). Indeed, the nanoparticles were placed in a plastic tube as a powder. The presence of the Lu atom led to X-ray absorption of BaLuF_5_: Yb^3+^-Er^3+^@Ethane with a good contrast observed as already seen with bare Lu-based nanospheres [19]. BaLuF_5_: Yb^3+^-Er^3+^@Ethane could thus be used as contrast agents for X-ray CT.

## 3. Materials and Methods

Cetyltrimethylammonium bromide (CTAB), sodium hydroxide, ammonium nitrate (NH_4_NO_3_), and potassium bromide were purchased from Sigma-Aldrich. Absolute ethanol was purchased from Fisher Chemicals and hydrochloric acid from VWR PROLABO. Bis(triethoxysilyl)ethane and bis(triethoxysilyl)ethylene were purchased from ABCR (Karlsruhe, Germany).

Scanning electron microscope (SEM): Hitachi S4800 (Krefeld, Germany). The qualitative composition of the particles was assessed by energy dispersive X-ray analysis (EDX, EDX Bruker X Flash Detector 4010, Krefeld, Germany) coupled with the SEM microscope. TEM images were recorded with a JEOL 1200 EXII microscope (JEOL Europe SAS, Croissy Sur Seine, France) to visualize the shape of the NPs, HRTEM observation was performed with MET FEG JEOL 2200 FS, 200 kV ((JEOL Europe SAS, Croissy Sur Seine, France). The element analysis on the observed core-shell nanoparticles (BaLuF_5_: Yb^3+^-Er^3+^@Ethane) was performed with DX TEM JEOL 1400 P+, 120 kV, and EDX SDD OXFORD. The presence of other elements such as copper or gold is due to the TEM grids and from the grid holder. For the purpose of TEM observations, the sample particles were dispersed in ethanol and then dropped onto copper grids with porous carbon films. The specific area and pore structure parameters of the studied NPs were determined from the measurements of nitrogen adsorption–desorption at 77 K with a TRISTAR 3000 Micromeritics using the Brunauer–Emmet–Teller (BET) method. For the sorption experiments, the samples (about 70 mg) were evacuated under vacuum at 80 °C for 12 h. DLS were recorded with a Vasco 135 Cordouan apparatus (Cordouan Technologies, Pessac, France).

Luminescence: The excitation is provided by a 980 nm beam of diode laser CNI Model FC-980 nm-4 W (cnilaser, Changchun, China). The UC emitted photons arising from NaYF_4_:Yb^3+^, Er^3+^@Ethane in ethanol are detected at room temperature at a right angle from the excitation and analyzed through a Jobin-Yvon monochromator (Triax 550, HORIBA Jobin Yvon, Palaiseau, France) combined with a cryogenically cold charge-coupled device (CCD) camera (Jobin-Yvon Symphony LN2 series, HORIBA Jobin Yvon, Palaiseau, France).

Synthesis of NaYF_4_:18%Yb^3+^, 2%Er^3+^ nanoparticles: YCl_3_ (0.8 mmol), YbCl_3_ (0.18 mmol) and ErCl_3_ (0.02 mmol) were dissolved in a mixture of 6 mL of oleic acid and 15 mL of octadecene (3/5 *v*/*v*). The solution was brought to 160 °C for 1 h to obtain a homogeneous mixture and the solution was cooled to room temperature. A second solution, containing 2.5 mmol of NaOH and 4 mmol of NH_4_F previously dissolved in 10 mL of methanol, was added slowly to the previous mixture. The whole mixture was stirred for 30 min. and then heated at 100 °C for 10 min. Finally, the temperature was set at 320 °C for 1 h under argon flow. The solution was then cooled to room temperature and the nanocrystals were precipitated with ethanol. The nanocrystals were then washed with a cyclohexane/ethanol mixture (1/1 *v*/*v*) three times.

Synthesis of BaLuF_5_:20%Yb^3+^-2%Er^3+^ nanoparticles: Appropriate amounts of barium nitrate (Ba(NO_3_)_2_), lutetium acetate hydrate ((CH_3_CO_2_)_3_Lu·xH_2_O), ytterbium acetate hydrate ((CH_3_CO_2_)_3_Yb·xH_2_O), and erbium acetate hydrate ((CH_3_CO_2_)_3_Er·xH_2_O) were dissolved in 9.5 mL of ethylene glycol and admixed with 0.5 mL of 1-butyl-3-methylimidazolium tetrafluoroborate ([BMIM]BF_4_). The resulting solution (final concentration of Ba = 0.01 M, Lu = 0.01 M, Yb = 2 ×·10^−3^ M, Er = 2 × 10^−5^ M) was magnetically stirred for 5 min at room temperature to favor homogenization and aged for 15 h. in tightly closed test tubes using an oven preheated at 120 °C. After cooling down to room temperature, the suspension was centrifuged to remove supernatant and washed with ethanol and water.

Synthesis of BaLuF_5_: Yb^3+^-Er^3+^@Ethylene: CTAB (38.4 mg) and NaOH (1 M, 78 µL) were mixed in H_2_O (120 mL), and heated at 80 °C at a stirring speed of 750 rpm. In a separate vial, BaLuF_5_: Yb^3+^-Er^3+^ nanoparticles (1 mL) were diluted in H_2_O (1 mL) and sonicated for 1 h. The BaLuF_4_ nanoparticles were then added in the surfactant solution and stirred for 30 min. at 750 rpm. Bis(triethoxysilyl)ethylene (200 µL, 0.53 mmol) in 18 mL EtOH was then added to the solution and the reaction stirred at 80 °C for 2 h. The reaction was cooled to RT, then centrifuged (20 min, 20,000 rpm), and the surfactant was extracted with a solution of NH_4_NO_3_ (6g L^−1^; EtOH 95%), by dispersing the nanoparticles at 40 °C under ultrasound for 20 min followed by centrifugation. The procedure was repeated twice, and the nanoparticles washed with water and EtOH. The powder was dried at RT under vacuum.

Synthesis of BaLuF_5_: Yb^3+^-Er^3+^@Ethane: CTAB (125 mg) and NaOH (2 M, 437 µL) were mixed in H_2_O (60 mL), and heated at 80 °C at a stirring speed of 750 rpm. In a separate vial, BaLuF_5_: Yb^3+^-Er^3+^ nanoparticles (1.5 mL) were diluted in H_2_O (1 mL) and sonicated for 1 h. The BaLuF_5_ nanoparticles were then added in the surfactant solution and stirred for 30 min. at 750 rpm. Bis(triethoxysilyl)ethane (184 µL, 0.49 mmol) was then added to the solution and the reaction stirred at 80 °C for 2 h. The reaction was cooled to RT, then centrifuged (20 min, 20,000 rpm), and the surfactant was extracted with a solution of NH_4_NO_3_ (6g L^−1^; EtOH 95%), by dispersing the nanoparticles at 40 °C under ultrasounds for 20 min. and centrifugation. The procedure was repeated twice, and the nanoparticles washed with water and EtOH. The powder was dried at RT under vacuum.

Synthesis of NaYF_4_: Yb^3+^-Er^3+^@Ethane: CTAB (125 mg), NaOH (2 M, 437 µL), were mixed in H_2_O (60 mL), and heated at 80 °C at a stirring speed of 750 rpm. In a separate vial, NaYF_4_: Yb^3+^-Er^3+^ nanoparticles (16 mg) were diluted in H_2_O (2 mL) and sonicated for 1 h. The NaYF_4_ nanoparticles were then added in the surfactant solution and stirred for 30 min at 750 rpm. Bis(triethoxysilyl)ethane (184 µL, 0.49 mmol) was then added to the solution and the reaction stirred at 80 °C for 2 h. The reaction was cooled to RT, then centrifuged (20 min, 20,000 rpm), and the surfactant was extracted with a solution of NH_4_NO_3_ (6g L^−1^; EtOH 95%), by dispersing the nanoparticles at 40 °C under ultrasound for 20 min followed by centrifugation. The procedure was repeated twice, and the nanoparticles washed with water and EtOH. The powder was dried at RT under vacuum.

Cell culture: Human breast adenocarcinoma cell line (MCF-7) were maintained in Dulbecco’s Modified Eagle Medium: Nutrient Mixture F-12 (DMEM/F12), which is a commercial medium widely used for the culture of mammalian cells and available in all consumable providers for cell biology. Additionally, 10% of fetal bovine serum was added for proteins, fatty acids, and growth factors. This medium was also supplemented with antibiotics (50 μg·mL^−1^ gentamycin) to ensure cell safety. Finally, these cells were allowed to grow in a humidified atmosphere at 37 °C under 5% CO_2_.

Cytotoxicity study with NaYF_4_: Yb^3+^-Er^3+^@Ethane: For cell viability assay, cells were seeded in 96-well plate in 200 µL of their respective culture medium. 24 h after cell growth; cells were treated with different concentrations of nanoparticles and incubated for 3 days. After the incubation time, cells were incubated for 3 h with 0.5 mg mL^−1^ of MTT (3-(4,5-dimethylthiazol-2-yl)-2,5-diphenyltetrazoliumbromide). Then, the MTT/medium was removed and the precipitated crystals were dissolved in ethanol/DMSO (1:1) solution with shaking for 20 min. The absorbance was measured at 540 nm.

The dose-response curve was plotted as the log concentration (µg mL^−1^) versus the percentage of cell viability. The mean lethal concentration (LC50) was determined by a non-linear regression equation using GraphPad Prism 5.0 software (San Diego, CA, USA).

Fluorescence imaging of cell uptake of NaYF_4_: Yb^3+^-Er^3+^@Ethane: Human breast cancer cells MCF-7 were seeded into a tissue culture chamber with a cover glass bottom in 300 µL of culture medium. Then, the cells were incubated for 24 h with NaYF_4_: Yb^3+^-Er^3+^@Ethane at a concentration of 50 µg·mL^−1^. Fifteen minutes before the end of incubation, cells were loaded with Cell Mask (Invitrogen, Cergy Pontoise, France) for membrane staining at a final concentration of 5 μg mL^−1^ The cells were washed twice with 1 mL of culture medium. Fluorescence imaging (980 nm excitation wavelength) was performed on living cells with a Carl Zeiss two-photon confocal microscope.

Cellular uptake quantification of NaYF_4_: Yb^3+^-Er^3+^@Ethane: The cellular uptake experiment was performed using confocal fluorescence microscopy on living cells. MCF-7 cells were plated on Lab-Tek II Chambered Coverglass (Nunc Ref 155382, Waltham, MA, USA) in a 0.5 mL culture medium for 24 h. Then, cells were treated with concentrations lower than LC50, 25 µg mL^−1^ and 50 µg mL^−1^, for 24 h. Control cells were treated with vehicle. Fifteen minutes before the end of incubation, nuclei were stained with Hoechst 33,342 at a final concentration of 5 μg mL^−1^. Then cells were washed two times with culture medium. Confocal fluorescence microscopy was performed on living cells under a 980 nm wavelength excitation for nanoparticles, 750 nm for nuclei and 522 nm for cell membranes. To calculate the percentage of labeled cells with nanoparticles, cells were counted using the ImageJ program and values obtained were the means of 9 pictures for each concentration.

X-ray computed tomography: X-ray computed tomography images were recorded on the BaLuF_5_: Yb^3+^-Er^3+^@Ethane nanoparticles as a powder in a plastic tube showing a transversal image, evidenced that the nanoparticles significantly absorbed the X-ray photons since the image exhibits a suitable contrast. Micro-CT (eXplore CT120; GE HealthCare, Buc, France) examinations were performed using the following parameters: the acquisition consisted of the 720 views acquired, with a 20 ms exposure time per view (Gain: 25 and Offset: 20). The X-ray tube voltage and current were set at 100 kV and 50 mA. CT images were reconstructed using a binning mode of 1 × 1 × 1 and a voxel size of 50 × 50 × 50 µm^3^. Images were analyzed using MicroView analysis + software (version 2.2, GE Healthcare, Buc, France).

## 4. Conclusions

In conclusion, we studied the syntheses of core-shell systems with upconversion nanoparticles as their core and with a periodic mesoporous organosilica shell. Depending on the precursor used (bis(triethoxysilyl)ethane or bis(triethoxysilyl)ethylene), the upconversion core nanoparticles were either wrapped with the mesoporous organosilica layer or located on the top of the organosilica nanorods. The NaYF_4_: Yb^3+^-Er^3+^@Ethane and BaLuF_5_: Yb^3+^-Er^3+^@Ethane nanosystems presented interesting features for theranostics applications such as imaging upon near-infrared or X-ray excitation respectively.
